# Issues Faced by Pharmacy Technicians While Maintaining Automated Dispensing Cabinets and How to Overcome Them in the National Guard Health Affairs in Riyadh: A Qualitative Study

**DOI:** 10.7759/cureus.42210

**Published:** 2023-07-20

**Authors:** Asma A Alzahrani, Thamer M Aledresee, Ali M Alzahrani

**Affiliations:** 1 Department of Health Informatics, College of Public Health and Health Informatics, King Saud Bin Abdulaziz University for Health Sciences, Riyadh, SAU; 2 Department of Pharmaceutical Care, King Abdulaziz Medical City, National Guard Health Affairs, Riyadh, SAU; 3 Oncology Center, King Fahad Medical City, Riyadh, SAU

**Keywords:** qualitative study, pharmacy technicians, pharmacy, automated dispensing cabinets, adc

## Abstract

Objectives

Technology is rapidly evolving to improve patient safety and increase healthcare providers’ efficiency. Automated dispensing cabinets (ADCs) are an example of a technology that has been used to facilitate patient safety. As with any other technology, there are benefits and drawbacks associated with the use of ADCs. In this study, we aim to identify the issues related to maintaining ADCs in National Guard Health Affairs (NGHA) hospitals from the pharmacy technicians’ perspective and find some solutions to overcome the problems that complicate the usability of the ADCs.

Methods

A cross-sectional qualitative study was conducted using an open-ended questionnaire. It was completed by 30 pharmacy technicians who deal with ADCs in NGHA hospitals.

Results

Three themes were extracted from the questionnaire: “issues faced by pharmacy technicians before filling the ADCs,” “issues faced by pharmacy technicians during filling the ADCs,” and “issues faced by pharmacy technicians after filling the ADCs.”

Discussion and conclusion

This study portrayed a better understanding of the issues faced by pharmacy technicians who deal with ADCs based on their experience. It will help stakeholders to make appropriate decisions and improve the workflow for a successful ADC implementation.

## Introduction

In 1999, the Institute of Medicine published a report called "To Err is Human: Building a Safer Health System." The report caught the eyes of the media, news, and the public because of the shocking number of injuries and deaths related to medical errors, which were estimated to be 98,000 hospital deaths per year [1,2]. The report stated that the leading cause of these medical errors is medication prescribing errors and adverse drug events. Medication errors were the reason behind 7,391 deaths in the US in 1993 [3]. Therefore, technology is rapidly evolving to improve patient safety and increase healthcare providers’ efficiency [3]. Automated dispensing cabinets (ADCs) are an example of a technology used to facilitate patient safety [4].

Automated dispensing cabinets

We can define ADCs as decentralized, computer-controlled systems used to store, distribute, and track medications at the point of care in the wards; they also provide a way to control the inventory [4-6]. ADCs are also known as unit-based cabinets (UBC), automated dispensing devices (ADD), and automated dispensing machines (ADM) [7-9]. There are many types of ADCs; some consist of drawers, called drawer modules, and others consist of shelves, called tower modules. Shelves are usually used for bulk medications, while drawers are used for unit-dose medications. Some other modules have drawers and shelves together to maximize their functionality [4,10].

ADCs are connected with the hospital's health information system (HIS) and the pharmacy information system (PIS) through Health Level 7 (HL7) standards. This interface between the systems allows the healthcare provider to use the ADC to see the patient medication list after the pharmacist revises it [4,11,12]. ADCs are usually filled with medications by pharmacy technicians. When the medication stock is low, the ADC will generate a report about the medications that need to be refilled and send it to the pharmacy [4].

The use of ADCs started in the late 1980s in US hospitals [5]. Adopting ADCs in hospitals was a prolonged process, but by 2007, more than 94% of US hospitals were using ADCs [5,13].

Benefits of using ADCs

The most important benefit of using ADCs in hospitals is improving patients’ safety by reducing medication errors [13]. ADCs can improve the medications’ distribution efficiency and administration by preventing any delay regarding medication availability, especially in emergencies [6,13,14]. Also, because ADCs interface with the pharmacy computer system, the pharmacist will review and check all the medications before administering them to the patients [5]. Overriding the pharmacist's medication-checking process is an available option in emergencies [13]. Another benefit of using ADCs in hospitals is that they help control and manage the medication inventory because their stock is tracked [4,5]. Moreover, ADCs can simply be a better way to store regular or controlled medications [15].

Challenges associated with using ADCs

One of the most common challenges regarding using any new technology is the difficulties end-users face when dealing with ADCs for the first time. This problem can be solved with sufficient training and follow-up assessments to ensure everyone uses the features of ADCs correctly [13,16]. Another matter is that only one healthcare provider can use the ADC at a time, and the others will have to wait in line for their turn; this could lead to errors like selecting the wrong medication [13]. ADCs should be located in a quiet space, perhaps a dedicated medication room away from any distractions. This might not be an option for every hospital, yet it is essential to prevent medication errors [13].

There is also the problem of misusing overrides, which happens when a healthcare provider takes medication from the ADC before the pharmacist reviews it. This might lead to medication errors like picking up the wrong medication from the ADC or administering the wrong dosage or form of medication. Override is a feature that should be used in emergencies only [5,13,17,18]. Picking up medication for more than one patient at a time is another opportunity for potential ADC misuse because this could cause a medication administration error [13]. There are also “look-alike medications,” and they should be stored in separate drawers to avoid mixing them up and causing medication errors [5,19].

Lastly, there is an interoperability problem between the ADCs and the pharmacy system. It is essential to maintain interoperability for pharmacists to review the medications before the nurses administer them [12].

ADCs in Saudi Arabia

There are few published papers about ADCs in Saudi Arabia [16]. One of those few papers was published in 2012, showing that only three hospitals in Riyadh were using ADCs integrated with the pharmacy system [16,20]. Another paper reported that in 2019, only 28.6% of Jeddah hospitals used ADCs [21]. This technology is relatively new here, yet the number of ADCs has increased over the years [11,16]. The research topics are limited and focused on measuring satisfaction with using ADCs and the improvements associated with implementing them in hospitals [16]. Accordingly, none of the previously mentioned papers focus on the difficulties associated with using ADCs in Saudi Arabia. Hence comes this study, which focuses on some of the issues related to operating and maintaining ADCs, especially from the point of view of pharmacy technicians.

The Ministry of National Guard Health Affairs (NGHA) has two hospitals in Riyadh: King Abdulaziz Medical City (KAMC) and King Abdullah Specialized Children's Hospital (KASCH). Both hospitals have a 1,796-bed capacity and around 68,564 in-patient admissions annually [22]. In 2017, NGHA started a project to install two different modules of ADCs to total 199 machines. They are manufactured by Omnicell® (Mountain View, CA) and distributed around the hospital in almost all the in-patient wards, including the intensive care units (ICUs) and regular wards. In this paper, we proposed a study to identify some of the issues faced by pharmacy technicians filling the ADCs and maintaining them in KAMC and KASCH in Riyadh. We aim to identify some solutions to improve the workflow in the zoning areas where pharmacy technicians prepare the ADCs’ stocks of medications.

## Materials and methods

A cross-sectional qualitative study was conducted over nine months, from March 2021 to December 2021, at NGHA hospitals in Riyadh. The research was conducted after obtaining the required official approval from King Abdullah International Medical Research Center’s (KAIMRC) Institutional Review Board (IRB) (approval number: SP21R/322/06). The study objectives were explained, and written consent was obtained before implementing the questionnaire. An open-ended questionnaire was used and distributed among the pharmacy technicians in the zoning areas, which are the areas where pharmacy technicians prepare the ADCs’ stock of medications (see Appendix for the informed consent form and the questionnaire). An open-ended questions questionnaire is more suitable for this study to determine some of the issues faced by the technicians while using and maintaining the ADCs and how to overcome them. The study was conducted in the zoning areas of KAMC and KASCH in Riyadh.

The study's population is the pharmacy technicians who work in the zoning areas in KAMC and KASCH. There are 68 pharmacy technicians working in those areas in KAMC and KASCH. The sample size is 30 pharmacy technicians, as was suggested in 2018 by Vasileiou et al. [23].

A purposeful sampling was conducted of the most experienced pharmacy technicians who deal with ADCs the most. A list of their names and years of experience was obtained from the zoning areas supervisors.

Statistical analysis

Descriptive statistics were used to summarize the pharmacy technicians’ demographic information. The rest of the data were analyzed using thematic analysis. All the collected data from the open-ended questions were coded and then combined and categorized into specific themes according to the six phases of reflexive thematic analysis developed by Braun and Clarke [24]. This approach was chosen because it is the most utilized and reliable approach; in addition, it has a clear framework that can be followed.

## Results

There were 30 participants in our study; their socio-demographic data are shown in Table 1. They answered our six open-ended questions regarding the issues they faced before, during, and after filling the ADCs. Their answers had been organized into themes, subthemes, and codes. There are three main themes. The first is the issues that the pharmacy technicians face before filling the ADCs and how to overcome them. The second theme concerns the pharmacy technicians' issues while supplying the ADCs and how to overcome them. In contrast, the third theme focuses on the pharmacy technicians’ problems after filling the ADCs and how to overcome them. Each theme has more than one subtheme. The first has two subthemes, i.e., issues regarding medication stock and staffing (Table 2). The second theme has four subthemes, i.e., issues regarding medication stock, issues regarding the workforce, issues regarding the barcode, and issues regarding the ADC (Table 3). There are two subthemes for the third theme, i.e., issues regarding the improper use of the ADC and regarding the workflow in the zoning area (Table 4).

**Table 1:** Pharmacy technicians’ socio-demographic information

Gender		Age (years)				Job position			Years of experience				
Female	Male	20 to 29	30 to 39	40 to 49	50 to 59	Technician III	Technician II	Technician I	Less than 5	5 to 10	11 to 15	16 to 20	21 to 25
24	6	2	12	14	2	12	10	8	2	16	5	6	1

**Table 2:** Issues faced by pharmacy technicians before filling the ADCs and how to overcome them ADCs: automated dispensing cabinets.

Theme	Subtheme	Codes (issues)	Frequency of the codes mentioned by participants	Codes (suggestions)	Frequency of the codes mentioned by participants
Issues faced by pharmacy technicians before filling the ADCs and how to overcome them	Issues regarding medications stock	Zero stock or not enough medications in the zoning area/pharmacy store/warehouse	21	Informing the planning department/repackaging center to prepare enough quantity ahead of time	15
				Trying to find the medication in the warehouse	1
				Proper endorsement to the pharmacists and physicians about the out-of-stock medications	1
	Issues regarding staffing	Medications are available but they need to be barcoded first	6	Hiring more staff/offering overtime opportunities to barcode the medications	4
				Printing enough barcode labels, especially for the fast-moving medications	1
				Preparing barcoded medications ahead of time	1
		Shortage of staff who barcode the medications	1	Hiring more staff/offering overtime opportunities	1

**Table 3:** Issues faced by pharmacy technicians during filling the ADCs and how to overcome them ADCs: automated dispensing cabinets; BUD: beyond-use date.

Theme	Subtheme	Codes (issues)	Frequency of the codes mentioned by participants	Codes (suggestions)	Frequency of the codes mentioned by participants
Issues faced by pharmacy technicians during filling the ADCs and how to overcome them	Issues regarding medications stock	Discrepancies between the actual number of medications in the ADC compared to the report that is automatically generated by the ADC and sent to the zoning area	27	Teaching nurses the proper way to use the ADC and the importance of deducting the medications they get from the ADC	12
				Making sure to fix the discrepancy before refilling the ADC	9
				Teaching all the healthcare personnel who have access to the ADC the proper way to use the ADC and the importance of deducting the medications they get from it	3
				Filling the medications in the presence of the nurses so they can see the effect of not deducting the medications taken from the ADC on the process of refilling	2
				Returning the excess medications to the zoning area	1
		The BUD of the medication does not match the BUD on the ADC screen	3	Correcting the BUD on the ADC screen before filling the medication in the cabinet or the drawer	3
		The presence of expired medications in the ADC drawers	2	Assigning one technician to be responsible for checking the medications’ expiry date in the ADCs	2
		The strength/volume of the available medications are not matching the ones registered in the ADC	1	Requesting from the automation team to edit/update the registered medications in the ADC to match the available stock	2
	Issues regarding workforce	Nurses’ interruptions while technicians are filling the ADC	13	Asking the nurses to cooperate and take the urgent (STAT) medications only since the filling process does not take more than 15 minutes	8
				Advising the nurses to take the medications they need before or after the filling process	2
				Having a fixed time for filling the ADC	1
		Some pharmacy technicians do not know how to fill the ADC in the proper way	1	Re-educating pharmacy technicians on how to fill the ADC properly	1
	Issues regarding the barcode	Hard to scan/unable to scan the barcode of some medications	6	Dedicating specific printers for printing barcode labels only or having a good-quality printer	3
		Some medications lose their barcode label	1	Re-printing the barcode label	1
	Issues regarding the ADC	The ADC machine is not working at the time of filling it	5	Assigning an on-call specialist to fix the ADC when needed	4
		Some of the ADC cabinets or drawers are small for the medication size or quantity	2	Having ADCs with bigger/adjustable cabinets or drawers	2

**Table 4:** Issues faced by pharmacy technicians after filling the ADCs and how to overcome them ADCs: automated dispensing cabinets.

Theme	Subtheme	Codes (issues)	Frequency of the codes mentioned by participants	Codes (suggestions)	Frequency of the codes mentioned by participants
Issues faced by pharmacy technicians after filling the ADCs and how to overcome them	Issues regarding the improper use of the ADC	Phone requests/blue slip requests of medications from the satellite pharmacies even though the ADC is refilled every day	8	Educating the nurses about the proper way to check and use the ADC before calling the pharmacy	7
				Pharmacists and technicians should check the reason for the request before supplying any ADC medications	1
		Some medications are requested by the nurses because they are fast moving and they run out on the same day	3	Increasing the fast-moving medications par level in the ADC by the automation team	3
		Phone calls from the nurses that the ADC is not allowing them to take the medication	2	Teaching the nurses how to use the ADC properly	1
		Receiving many emails from the nurses to refill some of the ADC medications (that are not included in the automatic report generated by the ADC due to discrepancy)	1	Teaching the nurses how to use the ADC and why it is important to deduct the number of medications they take from the ADC	1
	Issues regarding the workflow in the zoning area	Receiving emails from the nurses to refill some of the ADC medications (that are not included in the automatic report generated by the ADC due to discrepancy) after the technicians start their rounds to fill the ADCs	2	Educating the nurses about the importance of sending their refill emails ahead of time to allow the technicians to prepare the requested medications before they start their rounds	2

## Discussion

**Figure 1:**
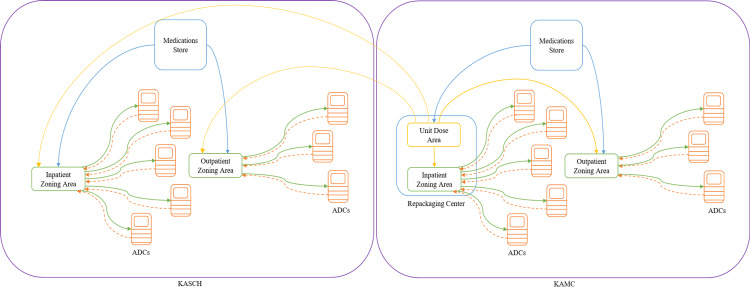
Overview of the workflow of filling the ADCs ADCs: automated dispensing cabinets; KASCH: King Abdullah Specialized Children's Hospital; KAMC: King Abdulaziz Medical City.

It was noticed that there are many complaints regarding ADCs in our hospitals, so we wanted to define these issues, especially those faced by pharmacy technicians who refill the ADCs. It will be helpful to start with an overview of the workflow for loading ADCs (Figure 1).

ADCs’ filling workflow

In NGHA hospitals, there are four zoning areas. Two cover the in-patient wards and ICUs, and the others cover the outpatient areas in KAMC and KASCH. In the zoning areas, pharmacy technicians receive automatically generated reports from the ADCs. These reports show the number of medications remaining in the ADC, like how many tablets of acetaminophen are present or how many milliliters there are of amlodipine syrup. The pharmacy technicians will use these reports to prepare the medications that each ADC needs and then do their rounds to fill each ADC in the hospital.

The ADCs are filled with unit dose medications prepared in the unit dose area in KAMC’s repackaging center. According to the American Society of Health-System Pharmacists (ASHP), the unit dose system is a medication distribution and dispensing method used in many organized healthcare settings. Unit dose medications like tablets, vials, or oral syrup are wrapped individually and barcoded. The medication name, strength, lot number, expiry date, and a barcode showing the same information after scanning are all printed on every unit dose package [25,26].

Medication stocks are provided to the unit dose area from the store in bulk before it is turned into unit dose form. Sometimes things do not go as planned, and there will be an issue here or there, which is why this study’s questionnaire was designed to determine these issues and find some solutions.

These issues have been grouped into three different themes as follows.

Issues before filling ADCs

From our pharmacy technicians’ experience, the most common problem they face before filling the ADCs is “there is not enough stock from the store and warehouse to fill the ADC,” shortage of quantity of some medications to supply the ADCs, or sometimes there is no stock at all. Drug shortage is a problem in many hospitals for different reasons. A study conducted by Mazer-Amirshahi et al. [27] found that the reasons behind critical care medications shortage are natural disasters, business decisions gone wrong, regulatory problems, shortage of the raw materials used in making medications, supply and demand problems, and finally, more than 50% of the problems are due to manufacturer-related challenges like delays.

The pharmacy technicians mentioned that informing the store beforehand about the quantity of each medication they need for each ADC might help to overcome this matter. Further, informing pharmacists and physicians about out-of-stock medications will encourage them to look for alternative medications in the short term.

Many medications in the bulk form need to be put in unit dose form and barcoded before the pharmacy technicians in the zoning area can use them to fill the ADC. This problem exists due to the staff shortage in the unit dose area. Alomi et al.'s [28] study published in 2019 reported the shortage of pharmacy technicians. Over the years from 2006 until 2017, the number of pharmacy technicians in Saudi Arabia increased, but the ratio of pharmacists to pharmacy technicians decreased. The study suggested that the optimal ratio is 1:4 in hospital settings [28]. Our pharmacy technicians think that offering overtime opportunities will help to solve this problem if hiring more pharmacy technicians is not feasible.

Issues during filling ADCs

The most common issue while filling ADCs is medication discrepancy. It can be defined as the difference between the actual number of medications in the ADC and the number registered in the system [29]. When pharmacy technicians do rounds to fill the ADCs, they find discrepancies between the actual number of medications in the ADCs’ bins and the report automatically generated by the ADCs and sent to the zoning area. Usually, the quantity of the medicines in the ADC’s bins or drawers is less than in the report. Still, one pharmacy technician reported otherwise, “Sometimes there is more medication in the bin than the par value level in the system.” The par level is the maximum number of unit dose medications that can be in each compartment in the ADC. It relies on the consumption level of the area the ADC is in, not on the space of the ADC’s compartment. According to her, the reason is that in case of emergencies, the nurses prefer to take the medication from the pharmacy directly without checking the ADC first. If it is an emergency, pharmacists will provide the medication immediately without instructing the nurses to check the ADC. Later, the medication might not be used and is returned to the ADC’s bin without correcting the medication quantity in the system.

In addition, it was noticed by our pharmacy technicians that medication discrepancies were more common among multidose vials like the Insulin or bulk liquid medications that were stored in the refrigerator outside the ADCs. The same problem was mentioned in a study performed in 2019 by Lichtner et al. [29], where discrepancies were more frequent in multidose liquid medications than in single-dose medicines. Sometimes nurses are adequately trained in using ADCs, but this does not seem to be the case among other healthcare personnel like respiratory therapists. It was also observed that there were discrepancies among medications for nebulization, like salbutamol and budesonide.

Our pharmacy technicians proposed the following to solve the medication discrepancy issue: pharmacy technicians need to fix the discrepancy in the system before refilling the medication bin or drawer, and they should take any excess medications back to the zoning area. There should be some education and training sessions for all the healthcare personnel who are using the ADCs on the proper way to use them and explaining the importance of deducting the number of the used medications from the ADC in the system. Training has a good effect on ADC users’ attitudes, as mentioned in a study by Elkady et al. [30] that was performed at King Faisal Specialist Hospital in Jeddah. The nurses’ attitude toward ADC use improved after a good training program [30]. Subsequently, there was a successful implementation process of ADCs in that hospital.

An interesting idea from one of the technicians regarding solving the discrepancy issue was using our safety reporting system (SRS) in the hospital. She wrote, “When we inform the nurses that we will issue an SRS once the quantity is not the same, they are more cautious; we must log the badge of the last nurse who got the medication that was not properly documented.” SRS is a tool that helps hospital workers to report any safety or quality issues for further investigation by the safety and quality department in the hospital. A similar suggestion was mentioned in Lichtner et al.’s [29] study, where they used the ADC to obtain a list of all the staff who had access to the discrepant medication to limit the scope of the investigation.

Another critical challenge that makes pharmacy technicians’ work difficult while they fill ADCs is nurses’ interruptions. Suppose it is time to dispense a medication, and the nurse wants to use the ADC to get it while technicians fill it. The pharmacy technicians say they do not need more than 15 minutes to fill one ADC. We registered quotes like, “Nurses should give us time to refill (15 minutes),” “Give us few minutes to finish filling the ADC,” and “If they can give us about five to 10 minutes to refill the ADC.” Nurses’ interruptions affect the accuracy of the medication count. “Technicians must be accurate in counting before refilling the medications.” To overcome this matter, our pharmacy technicians suggested a fixed time to do rounds and refill ADCs. Then, the nurses can take the medications they need before or after refilling without interruption unless it is an urgent (STAT) medication.

As stated before, the medication in a unit dose package is barcoded and has all the necessary information printed on it. It was observed that sometimes the barcode on the unit dose package could not be scanned or was hard to scan. This is a problem because having an unscannable barcode could lead to medication administration errors, as mentioned in a study by Zheng et al. [31]. Using suitable quality printers or dedicating a specific printer for only printing barcodes can solve this problem. Furthermore, sometimes the beyond-use date (BUD) printed on the unit dose package does not match the registered BUD on the ADC screen. This happens when two different batches of the same medication have different BUDs; one is older. All it takes to fix this issue is to correct the BUD on the ADC screen before filling the medication in the ADC’s cabinet or drawer.

Other issues were related to the ADC machine itself. Perhaps it is not working at the time of refilling, or some of its cabinets or drawers are too small for some of the medications’ sizes or quantities. This problem was mentioned in Dobson et al.'s [32] study; some of the ADC’s compartments are too small to fit some medication syringes. It is important to have an on-call specialist to fix the ADC when it is broken, especially during the weekends, and to have bigger or adjustable ADC compartments to fit all the medications’ sizes.

Additionally, there were uncommon issues mentioned by one to two pharmacy technicians: expired medications in the ADCs compartments, some pharmacy technicians lacking the knowledge to fill the ADC correctly, and some medicines losing their barcode labels, as with insulin vials. Moreover, the strength or volume of the available medications may not match the values registered in the ADC. For example, the available antacid medication is 150 ml bottle, but the one recorded in the ADC is 300 ml.

These problems can be remediated by the following: assigning one technician to be responsible for checking the medications’ expiry date in the ADCs; providing re-orientation sessions on how to fill the ADC correctly; re-printing the lost barcode labels; and communicating with the automation team to edit and update the registered medications in the ADC to match the available stock.

Issues after filling ADCs

Finally, we will discuss pharmacy technicians’ issues after filling ADCs. The one most frequently cited is that the satellite pharmacies receive many phone requests for ADC medications and many blue slips as well, even though all the ADCs are refilled every day. A quote was, “After refilling, we still receive blue slips for ADC medications.” The blue slip is an electronic request from the nurse to the pharmacy to refill any medication, and it is per patient. This situation happens for varied reasons; for example, the nurses do not know how to use the ADC, they are not aware that the medication they are requesting from the satellite pharmacy is an ADC item, and they cannot take the drugs from the ADC due to technical problems or simply because the medication is fast-moving and is already gone from the ADC.

It was suggested that to reduce the phone call requests and the blue slips, we need to “remind the end users (nurses) to always check the ADC before sending blue slips or calling the pharmacy.” Further, pharmacists and pharmacy technicians should check the reason behind the request before supplying any ADC medications to ensure the medication is needed. The automation team can increase the par level of the fast-moving drugs in the ADCs.

The satellite pharmacies receive many calls, and the zoning areas receive many emails to refill out-of-stock ADC medications. This is a quandary because if ADC users correctly deduct the medicines they are taking from the ADC, they would not need to send any emails. After all, the ADC automatically generates a report and sends it to the zoning area when there is a medication round to finish. Moreover, these emails get sent to the zoning areas after the pharmacy technicians have started their rounds at 10 am. Therefore, nurses need to wait until the next day to fulfill their requests, and in the meantime, they have to send blue slips to the satellite pharmacy for the out-of-stock medications. To get the supply of drugs from the zoning area smoothly, “the nurses should deduct properly for whatever they take from the ADC” and send their refill requests before rounds start.

Study limitations

Research with a larger sample size from multiple hospitals is needed to have more diverse points of view from different pharmacy technicians who might have contrasting experiences on how to deal with ADCs. Further studies are recommended to get more information regarding the maintenance of ADCs in other hospitals.

## Conclusions

In conclusion, such errors are harmless to the patient, but it is important to address them since they affect the quality of workflow and the success of implementing ADCs. The first step toward solving any problem is addressing it, which we did in this research. We listed all the issues faced by our pharmacy technicians who deal with ADCs. Three themes were extracted, and each one has multiple subthemes: “issues faced by pharmacy technicians before filling the ADCs,” “issues faced by pharmacy technicians during filling the ADCs,” and “issues faced by pharmacy technicians after filling the ADCs.” In addition, we mentioned some of what we think might help solve these issues to improve the workflow of the pharmacy technicians and the attitudes of ADC users. Hopefully, there will be fewer complaints and more satisfaction from all the staff who deal with ADCs in our hospitals.

## Figures and Tables

**Figure 2:**
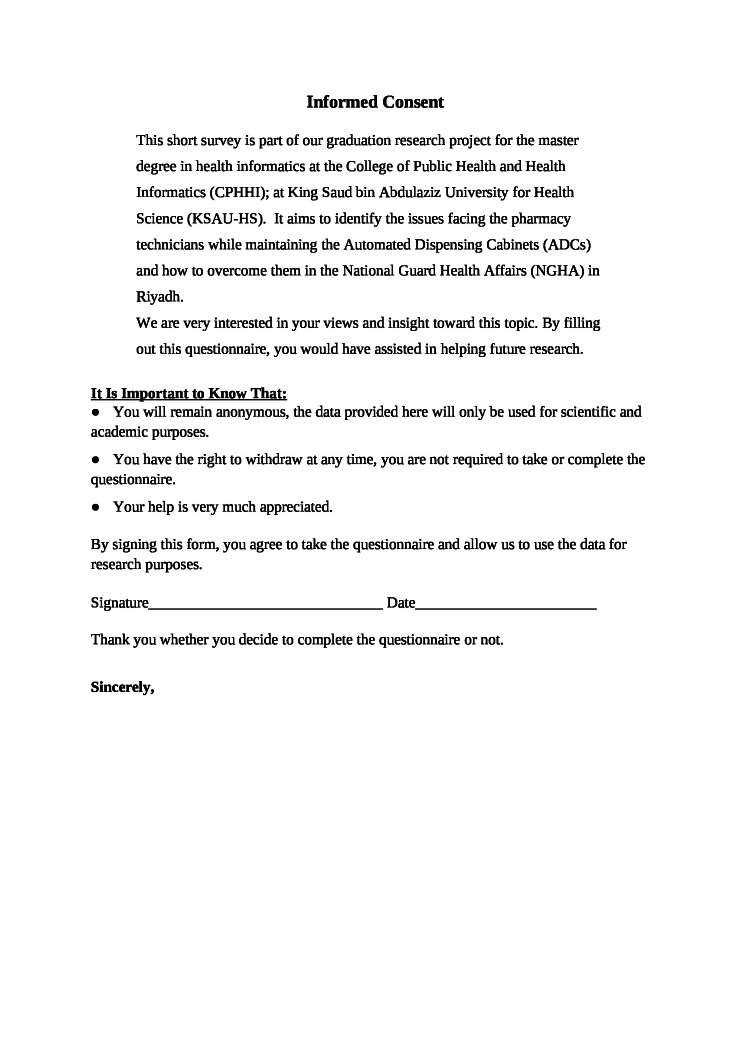
Informed consent form

**Figure 3:**
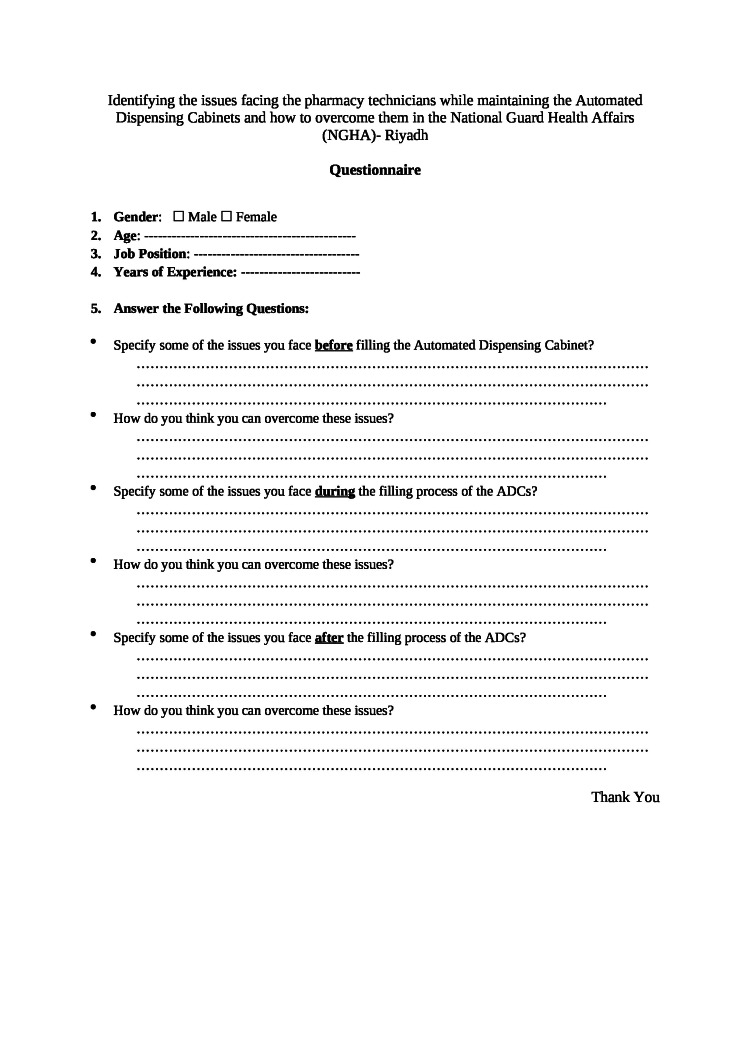
Questionnaire
